# Culture-Independence for Surveillance and Epidemiology

**DOI:** 10.3390/pathogens2030556

**Published:** 2013-09-24

**Authors:** Benjamin C. Kirkup

**Affiliations:** 1 Department of Wound Infections, Walter Reed Army Institute of Research, Silver Spring, MD 20910, USA; E-Mail: benjamin.kirkup@us.army.mil; Tel.: +301-319-3140; 2 Uniformed Services University of the Health Sciences, Bethesda, MD 20914, USA

**Keywords:** culture-independence, next generation sequencing, diagnostics

## Abstract

Culture-independent methods in microbiology (quantitative PCR (qPCR), sequencing, microarrays, direct from sample matrix assisted laser desorption/ionization time of flight mass spectroscopy (MALDI-TOF MS), *etc*.) are disruptive technology. Rather than providing the same results as culture-based methods more quickly, more cheaply or with improved accuracy, they reveal an unexpected diversity of microbes and illuminate dark corners of undiagnosed disease. At times, they overturn existing definitions of presumably well-understood infections, generating new requirements for clinical diagnosis, surveillance and epidemiology. However, current diagnostic microbiology, infection control and epidemiology rest principally on culture methods elegantly optimized by clinical laboratorians. The clinical significance is interwoven; the new methods are out of context, difficult to interpret and impossible to act upon. Culture-independent diagnostics and surveillance methods will not be deployed unless the reported results can be used to select specific therapeutics or infection control measures. To cut the knots surrounding the adoption of culture-independent methods in medical microbiology, culture-dependent methods should be supported by consistent culture-independent methods providing the microbial context. This will temper existing biases and motivate appropriate scrutiny of the older methods and results.

## 1. Introduction

### 1.1. The Advent of Culture Independence

Culture-independent methods have begun to transition from environmental microbiology, where axenic culture is recognized to yield only 1% of bacteria [1], to diagnostic microbiology, where culture continues to be a significant element of the accepted standard (Manual of Clinical Microbiology [2]; itself a very forward-looking text, particularly in Section 1, which includes both current practice and commentary on the human microbiome and microbial genomics). In environmental microbiology, the progress of pure culture methods was paralleled by the progress of enrichment cultures [3,4], reminding researchers of the presence of uncultured organisms. Continuous efforts to enlarge the realm of the culturable were tedious and trying. Microscopy [5] and other direct detection methods continued to be used despite significant labor requirements and the difficulties of identifying organisms from morphology and staining alone [6]. Use of these methods to confirm the quantification of supposedly culturable organisms revealed the “great plate count anomaly” [7], which suggested that even many organisms thought to be effectively cultured could not, in fact, be accurately detected or quantified in conventional culture.

While environmental microbiology was forced to utilize difficult methods, because so few organisms could be cultured axenically, clinical microbiology continued to develop an admirable efficiency and cost effectiveness. Routine methods were continuously streamlined; such that in 1977, just as rRNA sequencing was entering use in microbial ecology and evolution [8], only 13 of 38 hospital laboratories were found to include the main culture-independent method, the Gram stain, in microbiological sputum examination [9]. As published in the Journal of Clinical Microbiology, this led to a confirmation bias; the organisms thought to be most common were indeed found most commonly, and culture biases were reinforced. Further, even as more advanced methods, such as phenotype arrays and whole genome sequencing (WGS; *i.e.*, pathogenomics), were used to characterize isolated strains, the methods were necessarily applied to the cultured isolates. 

The advent of nucleic acid (“molecular”) methods has opened up the floodgates in environmental microbiology [10]. Vast microbial diversity, always apparent, has become tangible. Sequencing of diverse ribosomal RNA from communities provides a nearly open ability to identify bacteria and fungi with relative quantification [11]. Shotgun metagenomic sequencing provides more comprehensive information about identity and function [12]. Advanced microscopy provides single-cell data about genetics and physiology within populations and communities. Unexpected diversity was uncovered in the form of new and unexpected genera, as well as unexpected diversity within species and phenotypic plasticity within strains. Culture-independent methods have now been brought to bear on human-associated microbial flora (the “human microbiome”) in the contexts of apparent health and clinical disease. The results have been well publicized, (“The Gut Microbiota [Special Issue]” [13], for example). The microbial communities required for health are perhaps more diverse than expected and, at the same time, less so than was feared [14]. The newly available benchmark for this unexplored microbial diversity pushes the scientific community toward greater efforts at bringing uncharacterized organisms into culture and allows an assessment of relative success [15,16,17].

As the databases created from relatively open-ended culture-independent methods, such as high-throughput sequencing, expand, the opportunity to effectively apply closed methods expands. Given a sufficiently large database developed through the systemic application of an open method, such as sequencing, these closed methods have the potential to reveal the full diversity of microbes present in a sample, despite the theoretical limitation on the ability to detect or characterize a “never seen before” organism. Quantitative PCR [18,19], Nanostring [20] or microarray hybridization [21,22] and multiplex fluorescence *in situ* hybridization (FISH) [23] provide the ability to quantify taxa through their nucleic acid signatures and may be directly applied to samples (not isolates) to quantify an immense diversity of uncultured microbes directly from clinical samples without isolation. MALDI-TOF MS sensitively and specifically detects the ribosomal proteins; it has been applied to blood bottles [24,25]—culture without isolation—and may eventually be used directly on selected clinical samples [26]. Compared to sequencing, these closed methods each have advantages on the basis of cost, simplicity, sensitivity or speed.

### 1.2. The Clinical Relevance of Microbial Diversity

The clinical importance of the previously unaccounted microbial diversity will be inevitably recognized. Current practice is most precise when one of a few clearly identified, readily cultivated pathogens cause a clearly delineated clinical presentation. In such cases, selective culture is a sensitive strategy for diagnostic confirmation, epidemiology and, possibly, environmental surveillance. In other clinical scenarios, the art of the microbiologist is sufficient to render a sophisticated interpretation of circumstantial laboratory evidence to the clinician supporting the current standard of care; this practical experience enables empiric therapy by formally and informally incorporating local and communal outcome data [27,28]. However, at the margins of clinical practice are uncultivable infections (such as orthopedic infections [29], otitis media [30] and brain abscesses [31,32]), obviously polymicrobial infections (such as chronic ulcers and oral infections [33]) and diseases of unknown cause (including intestinal inflammatory diseases). In each of these cases, arguments made about detecting extra-cellular DNA, non-viable organisms [34], “contaminants” [35] and “irrelevant” normal flora [36] are overcome by new data. Given the relevance of previously undetected flora to the clinical status of the patient, it is logically deduced that clinically practical culture methods do not provide accurate diagnostic data in these cases. 

Unfortunately, even when clinical culture misses a relevant fraction of the diversity and poorly enumerates the culturable organisms, the data is accepted uncritically, because of historical precedent, overwhelming volume and apparent internal consistency. The minority voice, suggesting that this data does not represent the whole picture, barely registers. There is no vibrant, antagonistic debate between those who understand and practice sophisticated culture-based microbiology and those who would advocate culture-independent microbiology; instead, there is a failure to communicate between those who understand both and those who have not thought deeply about either. The leading clinical microbiologists are themselves the most engaged in the efforts to redefine clinical practice, finding appropriate limits for the use of culture. To quote George Bernard Shaw, “Those who understand the steam engine and the electric telegraph spend their lives in trying to replace them with something better [37].”

## 2. The Example of Wound Infections

As an example of the difficulties caused by culture methods as presently applied, full-fledged hospital epidemiology studies of infections compare infection rates under different treatment conditions and attempt to relate the environmental presence of the relevant pathogens to the pathogens recovered from patients [38,39,40,41,42]. For a wound infection study, the supporting data is composed of three parts: wound infection rates, the identity of the bacteria isolated from the wounds and the identity and quantity of bacteria from environmental surveillance. Conceptually, these three data sets are sufficient to correlate bacteria in the hospital environment with the same bacteria causing wound infections in patients. However, the use of culture compromises the data.

### 2.1. Infection Rates

Current guides to diagnosing surgical site and soft-tissue wound infections (wound assessment) still rely on the color of the wound bed, wound odor and exudate quantity/viscosity [43,44,45,46]. The most modern alternate is a subjective assessment of pain trends by the patient [47]. Subjective observations are almost self-evidently inadequate as diagnostic tools [48,49,50]; however, the alternative, clinical microbiology, is unable to distinguish infection from colonization or contamination of the wound, and experienced wound care practitioners routinely ignore culture results, if they request them in the first place [51,52].

### 2.2. The Identity of the Isolates from the Wounds

Clinical results reported in the literature are also unlikely to consistently identify and characterize “the wound pathogen”. Clinical microbiology for wound infections is typically a Gram stain and culture. The Gram stains and cultures typically are discordant when compared critically [53]. Submitted samples from physicians are usually swabs (recommendations from: Wounds UK, “Identifying Infection: Taking a Swab” [54]; and recent publications [55,56,57,58]). This sampling strategy is flawed [59,60,61], but remedying this is a low priority, because the downstream culture process is also ineffective. 

The clinical microbiologist typically selects a “representative” colony from the initial isolation, one not judged to be a “contaminant” or “commensal” by gross inspection of the Petri plate, and performs biochemical identification and antibiotic susceptibility tests. This down-selection to a single colony suppresses all interspecific and intraspecific diversity; also, various organisms previously considered to be “contaminants” are now known to have a role in infection [62,63,64,65,66,67,68,69,70,71,72,73,74]. In addition, contributing pathogens may not even grow on the plate; those that do may acquire specific “domestication” adaptations in the laboratory. Many wound organisms are functionally uncultured (slow to grow species, viable non-culturable, strict anaerobes, microaerophiles, requiring co-culture, *etc*.) [16,61,75,76,77,78]. The cultured organisms may not be numerous organisms [17]; and abundance may not always be an indication of relative pathogenic impact [79]. Whether the isolates are ultimately characterized biochemically or molecularly, relevant organisms are excluded by virtue of isolate collection. 

The final step of clinical microbiology is antibiotic susceptibility testing. However, in many cases, *in vitro* antibiotic susceptibility does not match *in vivo* clinical response to the antibiotics [80]. While there are some mechanistic reasons for this (biofilm formation, domestication adaptations, pharmacokinetics/pharmacodynamic variation, persisters, *etc*.), one overlooked issue is the probability that the primary pathogens were not the organisms tested for susceptibility. Ongoing debate in the wound care field challenges the clinical importance of the full microbial diversity, but recently, a clinical trial appears conclusive [76]. Recent results have not yet had their ultimate impact on the medical arts: treating infected wounds based on identification of all the organisms, culturable or not, enables wound healing in cases where amputation was judged largely inevitable.

### 2.3. The Identity and Quantity of Bacteria from Environmental Surveillance

The difficulty of environmental microbial sampling is well known by environmental microbiologists; culture-based microbiological surveys have, since the times of Beijerinck (1901) and Winogradsky (1890), been recognized as insufficient to study environmental microbial populations. However, they continue to be used by epidemiology and infection control programs [38,81,82,83,84], in large part due to a conviction that “medically relevant pathogens” are well-known and easily cultured. The current practices of pathogen surveillance mimic practices in clinical microbiology. Frequently, the methods are scrupulously imported from the clinical laboratory to surveillance; and the laboratory facilities, perhaps even the personnel, are shared. As in diagnostics, it is expensive and impractical to isolate all the colonies from a plate, colony morphology is used as a proxy for identity, “relevant” organisms are preferentially retained and culture is that obsidian lens through which all microbial diversity is filtered. Even molecular (PCR, pulse field gel electrophoresis (PFGE), sequencing) studies of “well-characterized” nosocomial pathogens [85,86,87,88] fundamentally rely on collection of isolates; while the relationships among isolates themselves may be well-understood, the underlying frequency and distribution data is untrustworthy.

In short, mutually reinforcing confirmation bias between clinical isolate statistics and surveillance statistics limits self-correction. The microbiological aspect of hospital epidemiology for wound infections is built on ineffective characterization of random subsamples from non-representative samples; when reading the literature, one can trust neither wound infection rate nor wound pathogen identification statistics (e.g., Guggenheim *et al*. 2009) [89]. The misrepresentation of pathogens is now known to occur in numerous clinical presentations [90,91,92,93,94,95,96]. It has been long recognized that the quality of the underlying data with regard to acute respiratory disease created a fetid myiasis of misinformation when compounded uncritically [97]. 

## 3. Hope and Change

Eventually, the expanded scientific literature will correct our misunderstandings of the microbial world; but presently, the cumulative culture-independent data are dwarfed by on-going culture-based pathogen surveillance. Volumes of historical data assert their relevance through mere existence. In addition, the continued use of culture is locked-in by the integration of culture-dependent diagnostics and the current generations of antibiotics. If a clinical condition (e.g., the presence of an uncultured organism in the context of certain signs and symptoms) is detectable only with culture-independent methods, specific therapeutics will not be developed until the diagnostics are deployed widely enough to stimulate demand. Meanwhile, culture-independent methods will not be deployed widely unless the data they generate can be used to select specific therapeutics—otherwise, it appears the money is wasted, because clinical decisions are unchanged. Antimicrobial development is lengthy [98,99] and the advance of sophisticated diagnostics too far ahead of therapeutics, presenting the specter of clinicians stoic in the face of infections understood, yet untreatable.

Fortunately, there are a number of on-going initiatives that will smooth the way forward towards a broadened view of infectious disease, including an appropriate interpretation of historical culture-biased data. Historically, culture-independent methods were costly, technically challenging, difficult to decipher and hard to compare across studies. The infectious disease community is well aware of the rapidly decreasing cost of producing culture-independent data [100]; it is perhaps increasingly aware of the vendors willing to perform microbial sequencing on raw samples (Second Genome, Inc., San Bruno, CA, USA; Mr. DNA, Research and Testing, Inc., Lubbock, TX, USA; and other certified service laboratories). Complementing the accessibility of data, high quality analysis is becoming manageable as the scientific community creates workflow management systems, pipelines and integrated packages. General purpose workflow management systems (Taverna [101], Galaxy [102], *etc*.) are available both for local installation and as web services. Integrated packages, such as MG-Rast [103], Qiime [104], AXIOME [105] and mothur [106], provide pre-defined and customizable, validated, published strategies for analyzing complex metagenome or community 16S rRNA data. While there is no substitute for an understanding of microbiology and molecular biology, these packages reduce the burden of the bioinformatics knowledge required to enter the field. In addition, several of these packages [107,108,109] are pre-configured to run on commercial computer systems (IaaS/PaaS or “cloud” computing), such as Amazon Web Services, so that the user does not need to have a significant investment in computing hardware and infrastructure, but is able to obtain highly responsive, timely results at a scalable cost. Costs for this strategy have been analyzed and published [110]. Finally, several tools are integrated with open data archives or platforms for sharing data in various stages of analysis (MG-Rast [103], HMP DACC [111,112], MoBeDAC, IMG [113]). This builds on the model of GenBank, which set a gold standard for data availability in the earlier years of sequencing. Open and easily accessible archives allow for integrated analyses, comparative studies, methods validation and re-analysis as new questions arise; and they are populated more quickly than data is published, in many cases, allowing for studies to build on each other ever more rapidly, with appropriate attribution.

Open data is a great innovation; open publication is also common in this particular research community. This has sped the availability of baseline data for the microbiome of humans, animals and the built environment. On-going projects and programs are producing baseline data for important collection sites, including every site on the human, in the home and throughout the hospital. The more rapid this accumulation of data and the more clinical and surveillance culture-independent data is available, the more meaningful new data becomes. 

## 4. Conclusions

Eventually, it should be possible to rehabilitate the culture-dependent literature, given an understanding of how the culturable fraction of microbes participates in and represents the larger microbial population from which it was drawn. Understanding weaknesses and confounders is critical to successfully utilizing the strengths of that literature. On the way to this vision, it is necessary to describe that larger microbial population and tie it to accurate diagnostic data, attentive to all the organisms in the patient or environment. In the process of collecting samples from environmental and clinical samples, molecular data is required to identify both the background assemblage of microbes in the sample, as well as to raise awareness of any additional strains of the same species, which are potentially important in epidemiology and pathogenesis [114,115], but may not be represented in culture or are a minority population not detected when single colonies are collected. 

In the interim, however, it is almost impossible to restrain the wealth of misinformation. Many readers, including administrators, policy makers and clinicians, are not afforded the time or subject matter expertise required to delve into the methods of each paper or report; they will not understand the issues inherent in conclusions presented, particularly when those conclusions concord with what was taught in schools, publicized in the press and was historically acceptable. “The difficulty lies, not in the new ideas, but in escaping from the old ones, which ramify, for those brought up as most of us have been, into every corner of our minds [116].”

In this case, the continuing flood of culture-biased data may slow progress towards enhanced medical care. Conscious efforts on the part of journal editors and subject matter experts are required to emphasize the limitations of data based on cultured isolate statistics and to encourage the inclusion of the culture-independent context whenever isolate data must be used for epidemiology, public health or infectious disease publication.

## References

[1] Wagner M., Amann R., Lemmer H., Schleifer K.H. (1993). Probing activated sludge with oligonucleotides specific for proteobacteria: Inadequacy of culture-dependent methods for describing microbial community structure. Appl. Environ. Microbiol..

[2] Versalovic J. (2011). Manual of Clinical Microbiology.

[3] Lester J.N., Perry R., Dadd A.H. (1979). Cultivation of a mixed bacterial population of sewage origin in the chemostat. Water Res..

[4] Ordal E.J., Palmer F.E., Malek I. (1964). Steady-state enrichment cultures of bacteria. Continuous Culture of Microorganisms.

[5] Kogure K., Simidu U., Taga N. (1979). A tentative direct microscopic method for counting living marine bacteria. Can. J. Microbiol..

[6] Austin B., Goodfellow M., Dickinson C.H. (1978). Numerical taxonomy of phylloplane bacteria isolated from Lolium perenne. J. Gen. Microbiol..

[7] Staley J.T., Konopka A. (1985). Measurement of in situ activities of nonphotosynthetic microorganisms in aquatic and terrestrial habitats. Annu. Rev. Microbiol..

[8] Woese C.R., Fox G.E. (1977). Phylogenetic structure of the prokaryotic domain: The primary kingdoms. Proc. Natl. Acad. Sci. USA.

[9] Heineman H.S., Chawla J.K., Lopton W.M. (1977). Misinformation from sputum cultures without microscopic examination. J. Clin. Microbiol..

[10] Allen E.E., Banfield J.F. (2005). Community genomics in microbial ecology and evolution. Nat. Rev. Microbiol..

[11] Amann R.I., Binder B.J., Olson R.J., Chisholm S.W., Devereux R., Stahl D.A. (1990). Combination of 16s ribosomal-RNA-targeted oligonucleotide probes with flow-cytometry for analyzing mixed microbial-populations. Appl. Environ. Microbiol..

[12] Schloss P.D., Handelsman J. (2005). Metagenomics for studying unculturable microorganisms: Cutting the Gordian knot. Genome Biol..

[13] (2012). Special Issue: The Gut Microbiota. Science.

[14] Fodor A.A., Desantis T.Z., Wylie K.M., Badger J.H., Ye Y., Hepburn T., Hu P., Sodergren E., Liolios K., Huot-Creasy H. (2012). The “most wanted” taxa from the human microbiome for whole genome sequencing. PLoS One.

[15] Goodman A.L., Kallstrom G., Faith J.J., Reyes A., Moore A., Dantas G., Gordon J.I. (2011). Extensive personal human gut microbiota culture collections characterized and manipulated in gnotobiotic mice. Proc. Natl. Acad. Sci. USA.

[16] Tuttle M.S., Mostow E., Mukherjee P., Hu F.Z., Melton-Kreft R., Ehrlich G.D., Dowd S.E., Ghannoum M.A. (2011). Characterization of bacterial communities in venous insufficiency wounds by use of conventional culture and molecular diagnostic methods. J. Clin. Microbiol..

[17] Shade A., Hogan C.S., Klimowicz A.K., Linske M., McManus P.S., Handelsman J. (2012). Culturing captures members of the soil rare biosphere. Environ. Microbiol..

[18] Smith C.J., Osborn A.M. (2009). Advantages and limitations of quantitative PCR (Q-PCR)-based approaches in microbial ecology. FEMS Microbiol. Ecol..

[19] Harris K.A., Hartley J.C. (2003). Development of broad-range 16S rDNA PCR for use in the routine diagnostic clinical microbiology service. J. Med. Microbiol..

[20] Barczak A.K., Gomez J.E., Kaufmann B.B., Hinson E.R., Cosimi L., Borowsky M.L., Hung D.T. (2012). RNA signatures allow rapid identification of pathogens and antibiotic susceptibilities. Proc. Natl. Acad. Sci. USA.

[21] Peter H., Berggrav K., Thomas P., Pfeifer Y., Witte W., Templeton K., Bachmann T.T. (2012). Direct detection and genotyping of *Klebsiella pneumoniae* carbapenemases from urine by use of a new DNA microarray test. J. Clin. Microbiol..

[22] Ballarini A., Segata N., Huttenhower C., Jousson O. (2013). Simultaneous quantification of multiple bacteria by the BactoChip microarray designed to target species-specific marker genes. PLoS One.

[23] Morgan M.A. (2013). Ten years of experience with peptide nucleic acid fluorescent in situ hybridization in the clinical microbiology laboratory. Clin. Microbiol. Newsl..

[24] Loonen A.J.M., Jansz A.R., Stalpers J., Wolffs P.F.G., van den Brule A.J.C. (2012). An evaluation of three processing methods and the effect of reduced culture times for faster direct identification of pathogens from BacT/ALERT blood cultures by MALDI-TOF MS. Eur. J. Clin. Microbiol. Infect. Dis..

[25] Chen J.H., Ho P.L., Kwan G.S., She K.K., Siu G.K., Cheng V.C., Yam W.C. (2013). Direct bacterial identification in positive blood cultures using two commercial MALDI-TOF mass spectrometry systems. J. Clin. Microbiol..

[26] Köhling H.L., Bittner A., Müller K.D., Buer J., Becker M., Rübben H., Mosel F. (2012). Direct identification of bacteria in urine samples by matrix-assisted laser desorption/ionization time-of-flight mass spectrometry and relevance of defensins as interfering factors. J. Med. Microbiol..

[27] Joo E.J., Kang C.I., Ha Y.E., Park S.Y., Kang S.J., Wi Y.M., Lee N.Y., Chung D.R., Peck K.R., Song J.H. (2011). Impact of inappropriate empiric antimicrobial therapy on outcome in *Pseudomonas aeruginosa* bacteraemia: A stratified analysis according to sites of infection. Infection.

[28] Davis M.E., Anderson D.J., Sharpe M., Chen L.F., Drew R.H. (2012). Constructing unit-specific empiric treatment guidelines for catheter-related and primary bacteremia by determining the likelihood of inadequate therapy. Infect. Control. Hosp. Epidemiol..

[29] Ehrlich G.D., DeMeo P., Palmer M., Sauber T.J., Altman D., Altman G., Stoodley P. (2012). Culture-negative infections in orthopedic surgery. Culture Negative Orthopedic Biofilm Infections.

[30] Ehrlich G.D., DeMeo P.J., Costerton J.W. (2012). The problem of culture-negative infections. Culture Negative Orthopedic Biofilm Infections.

[31] Al Masalma M., Armougom F., Scheld W.M., Dufour H., Roche P.H., Drancourt M., Raoult D. (2009). The expansion of the microbiological spectrum of brain abscesses with use of multiple 16S ribosomal DNA sequencing. Clin. Infect. Dis..

[32] Al Masalma M., Lonjon M., Richet H., Dufour H., Roche P.H., Drancourt M., Fournier P.E. (2012). Metagenomic analysis of brain abscesses identifies specific bacterial associations. Clin. Infect. Dis..

[33] Siqueira J.F., Rôças I.N. (2013). As-yet-uncultivated oral bacteria: Breadth and association with oral and extra-oral diseases. J. Oral Microbiol..

[34] Rogers G.B., Marsh P., Stressmann A.F., Allen C.E., Daniels T.V., Carroll M.P., Bruce K.D. (2010). The exclusion of dead bacterial cells is essential for accurate molecular analysis of clinical samples. Clin. Microbiol. Infect..

[35] Yao G.J., Gong J., Zhang G., Li C.C., Liu Z., Du W., Xu G., Wei K. (2012). Resolution of intracerebral *Bacillus cereus* infection following open neck injury after comprehensive treatment. Afr. J. Microbiol. Res..

[36] Sontakke S., Cadenas M.B., Maggi R.G., Diniz P.P.V.P., Breitschwerdt E.B. (2009). Use of broad range 16S rDNA PCR in clinical microbiology. J. Microbiol. Methods.

[37] Shaw G.B. (1903). Man and Superman.

[38] Rastogi S., Shah R., Perlman J., Bhutada A., Grossman S., Pagala M., Lazzaro M. (2012). Pattern of bacterial colonization in a new neonatal intensive care unit and its association with infections in infants. Am. J. Infect. Control.

[39] Weber D.J., Rutala W.A., Miller M.B., Huslage K., Sickbert-Bennett E. (2010). Role of hospital surfaces in the transmission of emerging health care-associated pathogens: *Norovirus*, *Clostridium difficile*, and *Acinetobacter* species. Am. J. Infect. Control.

[40] Samore M.H., Venkataraman L., DeGirolami P.C., Arbeit R.D., Karchmer A.W. (1996). Clinical and molecular epidemiology of sporadic and clustered cases of nosocomial *Clostridium difficile* diarrhea. Am. J. Med..

[41] Fawley W.N., Parnell P., Verity P., Freeman J., Wilcox M.H. (2005). Molecular epidemiology of endemic *Clostridium difficile* infection and the significance of subtypes of the United Kingdom epidemic strain (PCR ribotype 1). J. Clin. Microbiol..

[42] Ayliffe G.A. (1991). Role of the environment of the operating suite in surgical wound infection. Rev. Infect. Dis..

[43] Cutting K.F., White R.J. (2005). Criteria for identifying wound infection—Revisited. Ostomy Wound Manag..

[44] Cook L. (2011). Wound assessment: Exploring competency and current practice. Br. J. Community Nurs. Wound Care Suppl..

[45] Johnson and Johnson Company (2004). Applied Wound Management Assessment and Continuation Chart.

[46] Centers for Medicare and Medicaid Services (2012). OASIS-C.

[47] Reddy M., Gill S.S., Wu W., Kalkar S.R., Rochon P.A. (2012). Does this patient have an infection of a chronic wound?. JAMA.

[48] Falanga V., Grinnell F., Gilchrest B., Maddox Y.T., Moshell A. (1994). Workshop on the pathogenesis of chronic wounds. J. Invest. Dermatol..

[49] Robson M.C., Maggi S.P., Smith P.D., Wassermann R.J., Mosiello G.C., Hill D.P., Cooper D.M. (1999). Ease of wound closure as an endpoint of treatment efficacy. Wound Repair Regen..

[50] Patel G.K. (2007). How to diagnose and treat haemorrhagic skin necrosis. Wounds UK.

[51] Gardner S.E., Frantz R.A., Doebbeling B.N. (2001). The validity of the clinical signs and symptoms used to identify localized chronic wound infection. Wound Repair Regen..

[52] Moore K., Hall V., Paull A., Morris T., Brown S., McCulloch D., Richardson M.C., Harding K.G. (2010). Surface bacteriology of venous leg ulcers and healing outcome. J. Clin. Pathol..

[53] Kaftandzieva A., Cekovska Z., Kaftandziev I., Petrovska M., Panovski N. (2012). Bacteriology of wound—Clinical utility of gram stain microscopy and the correlation with culture. Maced. J. Med. Sci..

[54] Patten H. “Identifying wound infection: Taking a swab.”. http://www.wounds-uk.com/pdf/content_9492.pdf.

[55] Bonham P.A. (2009). Swab cultures for diagnosing wound infections: A literature review and clinical guideline. J. Wound Ostomy Cont..

[56] Gardner S.E., Frantz R.A., Saltzman C.L., Hillis S.L., Park H., Scherubel M. (2006). Diagnostic validity of three swab techniques for identifying chronic wound infection. Wound Repair Regen..

[57] Levine N.S., Lindberg R.B., Mason A.D., Pruitt B.A. (1976). The quantitative swab culture and smear: A quick, simple method for determining the number of viable aerobic bacteria on open wounds. J. Trauma Acute Care Surg..

[58] Lipsky B.A., Berendt A.R., Deery H.G., Embil J.M., Joseph W.S., Karchmer A.W., LeFrock J.L., Lew D.P., Mader J.T., Horden C. (2004). Diagnosis and treatment of diabetic foot infections. Clin. Infect. Dis..

[59] Angel D.E., Lloyd P., Carville K., Santamaria N. (2011). The clinical efficacy of two semi-quantitative wound-swabbing techniques in identifying the causative organism(s) in infected cutaneous wounds. Int. Wound J..

[60] Robson M.C. (1979). Infection in the surgical patient: An imbalance in the normal equilibrium. Clin. Plast. Surg..

[61] Kandula S., Zenilman J.M., Melendez J.H., Lazarus G.S., Sen C.K. (2010). New frontiers of molecular microbiology in wound healing. Advances in Wound Care.

[62] Dupont C., Sivadon‐Tardy V., Bille E., Dauphin B., Beretti J.L., Alvarez A.S., Carbonnelle E. (2010). Identification of clinical coagulase‐negative staphylococci, isolated in microbiology laboratories, by matrix‐assisted laser desorption/ionization‐time of flight mass spectrometry and two automated systems. Clin. Microbiol. Infect..

[63] Facklam R., Elliott J.A. (1995). Identification, classification, and clinical relevance of catalase-negative, gram-positive cocci, excluding the *streptococci* and *enterococci*. Clin. Microbiol. Rev..

[64] Facklam R. (2002). What happened to the *streptococci*: Overview of taxonomic and nomenclature changes. Clin. Microbiol. Rev..

[65] Huebner J., Goldmann D.A. (1999). Coagulase-negative *staphylococci*: Role as pathogens. Annu. Rev. Med..

[66] Khasriya R., Sathiananthamoorthy S., Ismail S., Kelsey M., Wilson M., Rohn J.L., Malone-Lee J. (2013). Spectrum of bacterial colonization associated with urothelial cells from patients with chronic lower urinary tract symptoms. J. Clin. Microbiol..

[67] Kleiner E., Monk A.B., Archer G.L., Forbes B.A. (2010). Clinical significance of *Staphylococcus lugdunensis* isolated from routine cultures. Clin. Infect. Dis..

[68] Kline K.A., Schwartz D.J., Gilbert N.M., Hultgren S.J., Lewis A.L. (2012). Immune modulation by group B *Streptococcus* influences host susceptibility to urinary tract infection by uropathogenic *Escherichia coli*. Infect. Immun..

[69] Klotchko A., Wallace M.R., Licitra C., Sieger B. (2011). *Staphylococcus lugdunensis*: An emerging pathogen. South. Med. J..

[70] Kobayashi K., Kami M., Ikeda M., Kishi Y., Murashige N., Tanosaki R., Takaue Y. (2005). Fulminant septicemia caused by *Bacillus cereus* following reduced-intensity umbilical cord blood transplantation. Haematologica.

[71] Nickel J.C., Xiang J. (2008). Clinical significance of nontraditional bacterial uropathogens in the management of chronic prostatitis. J. Urol..

[72] Papapetropoulos N., Papapetropoulou M., Vantarakis A. (2013). Abscesses and wound infections due to *Staphylococcus lugdunensis*: Report of 16 cases. Infection.

[73] Peters B.M., Jabra-Rizk M.A., Graeme A.O., Costerton J.W., Shirtliff M.E. (2012). Polymicrobial interactions: Impact on pathogenesis and human disease. Clin. Microbiol. Rev..

[74] Xu Y., Moser C., Al-Soud W.A., Sørensen S., Høiby N., Nielsen P.H., Thomsen T.R. (2012). Culture-dependent and-independent investigations of microbial diversity on urinary catheters. J. Clin. Microbiol..

[75] Wolcott R.D., Gontcharova V., Sun Y., Dowd S.E. (2009). Evaluation of the bacterial diversity among and within individual venous leg ulcers using bacterial tag-encoded FLX and titanium amplicon pyrosequencing and metagenomic approaches. BMC Microbiol..

[76] Dowd S.E., Wolcott R.D., Kennedy J., Jones C., Cox S.B. (2011). Molecular diagnostics and personalised medicine in wound care: Assessment of outcomes. J. Wound Care.

[77] Gontcharova V., Youn E., Sun Y., Wolcott R.D., Dowd S.E. (2010). A comparison of bacterial composition in diabetic ulcers and contralateral intact skin. Open Microbiol. J..

[78] Wolcott R.D., Gontcharova V., Sun Y., Zischakau A., Dowd S.E. (2009). Bacterial diversity in surgical site infections: Not just aerobic cocci any more. J. Wound Care.

[79] Percival S.L., Dowd S.E., Percival S., Cutting K. (2010). The microbiology of wounds. Microbiology of Wounds.

[80] Shin J.A., Chang Y.S., Kim H.J., Kim S.K., Chang J., Ahn C.M., Byun M.K. (2012). Clinical outcomes of tigecycline in the treatment of multidrug-resistant *Acinetobacter baumannii* infection. Yonsei Med. J..

[81] Brady R.R., Kalima P., Damani N.N., Wilson R.G., Dunlop M.G. (2007). Bacterial contamination of hospital bed-control handsets in a surgical setting: A potential marker of contamination of the healthcare environment. Ann. Roy. Coll. Surg..

[82] Goodman E.R., Platt R., Bass R., Onderdonk A.B., Yokoe D.S., Huang S.S. (2008). Impact of an environmental cleaning intervention on the presence of methicillin-resistant *Staphylococcus aureus* and vancomycin-resistant *enterococci* on surfaces in intensive care unit rooms. Infect. Control. Hosp. Epidemiol..

[83] Sutter D.E., Bradshaw L.U., Simkins L.H., Summers A.M., Atha M., Elwood R.L., Robertson J.L., Murray C.K., Wortmann G.W., Hospenthal D.R. (2011). High incidence of multidrug-resistant gram-negative bacteria recovered from Afghan patients at a deployed US military hospital. Infect. Control. Hosp. Epidemiol..

[84] Zhanel G.G., DeCorby M., Adam H., Mulvey M.R., McCracken M., Lagacé-Wiens P., Nichol K.A., Wierzbowski A., Baudry P.J., Tailor F. (2010). Prevalence of antimicrobial-resistant pathogens in Canadian hospitals: Results of the Canadian ward surveillance study (CANWARD 2008). Antimicrob. Agents Chemother..

[85] Dawson L.F., Valiente E., Donahue E.H., Birchenough G., Wren B.W. (2011). Hypervirulent *Clostridium difficile* PCR-ribotypes exhibit resistance to widely used disinfectants. PLoS One.

[86] Stabler R.A., Valiente E., Dawson L.F., He M., Parkhill J., Wren B.W. (2010). In-depth genetic analysis of *Clostridium difficile* PCR-ribotype 027 strains reveals high genome fluidity including point mutations and inversions. Gut Microbes.

[87] He M., Sebaihia M., Lawley T.D., Stabler R.A., Dawson L.F., Martin M.J., Holt K.E., Seth-Smith H.M., Quail M.A., Rance R. (2010). Evolutionary dynamics of *Clostridium difficile* over short and long time scales. Proc. Natl. Acad. Sci. USA.

[88] Stabler R.A., He M., Dawson L., Martin M., Valiente E., Corton C., Lawley T.D., Sebaihia M., Quail M.A., Rose G. (2009). Comparative genome and phenotypic analysis of *Clostridium difficile* 027 strains provides insight into the evolution of a hypervirulent bacterium. Genome Biol..

[89] Guggenheim M., Zbinden R., Handschin A.E., Gohritz A., Altintas M.A., Giovanoli P. (2009). Changes in bacterial isolates from burn wounds and their antibiograms: A 20-year study (1986–2005). Burns.

[90] Gadsby N.J. (2011). Evaluation of real-time 16S rDNA PCR and pyrosequencing for routine identification of bacteria in joint fluid and tissue specimens. Open J. Med. Microbiol..

[91] Sibley C.D., Church D.L., Surette M.G., Dowd S.E., Parkins M.D. (2012). Pyrosequencing reveals the complex polymicrobial nature of invasive pyogenic infections: Microbial constituents of empyema, liver abscess, and intracerebral abscess. Eur. J. Clin. Microbiol..

[92] Nelson C.L., McLaren A.C., McLaren S.G., Johnson J.W., Smeltzer M.S. (2005). Is aseptic loosening truly aseptic?. Clin. Orthop. Relat. R.

[93] Hoenders C.S., Harmsen M.C., van Luyn M.J. (2008). The local inflammatory environment and microorganisms in “aseptic” loosening of hip prostheses. J. Biomed. Mater. Res. B.

[94] Diaz R.R., Picciafuoco S., Paraje M.G., Villegas N.A., Miranda J.A., Albesa I., Cremonezzi D., Commisso R., Paglini-Oliva P. (2011). Relevance of biofilms in pediatric tonsillar disease. Eur. J. Clin. Microbiol..

[95] Saylam G., Tatar E.C., Tatar I., Ozdek A., Korkmaz H. (2010). Association of adenoid surface biofilm formation and chronic otitis media with effusion. Arch. Otolaryngol. Head Neck Surg..

[96] Liu C.M., Cosetti M.K., Aziz M., Buchhagen J.L., Contente-Cuomo T.L., Price L.B., Keim P.S., Lalwani A.K. (2011). The otologic microbiome: A study of the bacterial microbiota in a pediatric patient with chronic serous otitis media using 16SrRNA gene-based pyrosequencing. Arch. Otolaryngol. Head Neck Surg..

[97] Huebner R.J. (1957). Virologists dilemma. Ann. NY Acad. Sci..

[98] Jabes D. (2011). The antibiotic R and D pipeline: An update. Curr. Opin. Microbiol..

[99] Cooper M.A., Shlaes D. (2011). Fix the antibiotics pipeline. Nature.

[100] Loman N.J., Misra R.V., Dallman T.J., Constantinidou C., Gharbia S.E., Wain J., Pallen M.J. (2012). Performance comparison of benchtop high-throughput sequencing platforms. Nature Biotechnol..

[101] Oinn T., Addis M., Ferris J., Marvin D., Senger M., Greenwood M., Li P. (2004). Taverna: A tool for the composition and enactment of bioinformatics workflows. Bioinformatics.

[102] Goecks J., Nekrutenko A., Taylor J., Team T.G. (2010). Galaxy: A comprehensive approach for supporting accessible, reproducible, and transparent computational research in the life sciences. Genome Biol..

[103] Meyer F., Paarmann D., D’Souza M., Olson R., Glass E.M., Kubal M., Edwards R.A. (2008). The metagenomics RAST server—A public resource for the automatic phylogenetic and functional analysis of metagenomes. BMC Bioinformatics.

[104] Caporaso J.G., Kuczynski J., Stombaugh J., Bittinger K., Bushman F.D., Costello E.K., Knight R. (2010). QIIME allows analysis of high-throughput community sequencing data. Nat. Methods.

[105] Lynch M.D., Masella A.P., Hall M.W., Bartram A.K., Neufeld J.D. (2013). AXIOME: Automated exploration of microbial diversity. GigaScience.

[106] Schloss P.D., Westcott S.L., Ryabin T., Hall J.R., Hartmann M., Hollister E.B., Weber C.F. (2009). Introducing mothur: Open-source, platform-independent, community-supported software for describing and comparing microbial communities. Appl. Environ. Microbiol..

[107] Afgan E., Baker D., Coraor N., Goto H., Paul I.M., Makova K.D., Taylor J. (2011). Harnessing cloud computing with Galaxy Cloud. Nat. Biotechnol..

[108] Angiuoli S.V., Matalka M., Gussman A., Galens K., Vangala M., Riley D.R., Fricke W.F. (2011). CloVR: A virtual machine for automated and portable sequence analysis from the desktop using cloud computing. BMC Bioinformatics.

[109] Ragan-Kelley B., Walters W.A., McDonald D., Riley J., Granger B.E., Gonzalez A., Caporaso J.G. (2013). Collaborative cloud-enabled tools allow rapid, reproducible biological insights. ISME J..

[110] Angiuoli S.V., White J.R., Matalka M., White O., Fricke W.F. (2011). Resources and costs for microbial sequence analysis evaluated using virtual machines and cloud computing. PLoS One.

[111] Peterson J., Garges S., Giovanni M., McInnes P., Wang L., Schloss J.A., Guyer M. (2009). The NIH human microbiome project. Genome Res..

[112] Wortman J., Giglio M., Creasy H., Chen A., Liolios K., Chu K., White O. (2010). A data analysis and coordination center for the human microbiome project. Genome Biol..

[113] Markowitz V.M., Chen I.M.A., Chu K., Szeto E., Palaniappan K., Jacob B., Kyrpides N.C. (2012). IMG/M-HMP: A metagenome comparative analysis system for the human microbiome project. PLoS One.

[114] Smati M., Clermont O., Le Gal F., Schichmanoff O., Jauréquy F., Eddi A., Denamur E., Picard B. (2013). Real-time PCR for quantitative analysis of human commensal *Escherichia coli* populations reveals a high frequency of sub-dominant phylogroups. Appl. Environ. Microbiol..

[115] Eyre D.W., Cule M.L., Griffiths D., Crook D.W., Peto T.E., Walker A.S., Wilson D.J. (2013). Detection of mixed infection from bacterial whole genome sequence data allows assessment of its role in *Clostridium difficile* transmission. PLoS Comput. Biol..

[116] Keynes J.M. (1936). The General Theory of Employment, Interest and Money.

